# Efficacy of Wen-Dan Decoction in the treatment of patients with coronary heart disease: A protocol for systematic review and meta-analysis

**DOI:** 10.1097/MD.0000000000028041

**Published:** 2022-01-07

**Authors:** Xiaoyu Zhang, Yingwei Wang, Lufei Liu, Hui Jiang, Jing Wang, Yang Xiao, Jianwei Wang

**Affiliations:** a Heilongjiang University of traditional Chinese Medicine, Harbin, China; b Heilongjiang Yongqing Institute of traditional Chinese Medicine, Harbin, China.

**Keywords:** coronary heart disease, systematic review, wendan decoction

## Abstract

**Background:**

Coronary heart disease (CHD) is a heart disease caused by myocardial ischemia, hypoxia or necrosis due to stenosis or occlusion of lumen caused by coronary atherosclerosis. It belongs to ischemic cardiomyopathy and is more common in clinic. Previous studies have shown that Wen-Dan Decoction (WDD) is safe and effective, but there is a lack of systematic reviews. The purpose of this study is to systematically study the efficacy of WDD in the treatment of patients with CHD.

**Methods:**

We will search the following databases: PubMed, EMBASE, Web of Science, Central, Chinese databases China Biomedical Literature, Wanfang Chinese digital periodical and conference database (Wanfang Database), China National Knowledge Infrastructure database, and VIP Chinese Science and Technique Journals Database (VIP) from inception to August 2021. All published randomized controlled trials related to this study will be included. The ongoing or unpublished trials will be searched from National Institutes of Health clinical registry Clinical Trials, International Clinical Trials Registry Platform and the Chinese clinical trial registration platform. Two researchers separately screened the literature and extracted data. The primary outcome is total effective rate. The RevMan V5.3 will be used to evaluate literature and data analysis synthesis.

**Results:**

This study will provide a reliable evidence-based basis for the clinical application of WDD in the treatment of patients with CHD.

**Conclusion:**

The effectiveness of WDD for CHD will be evaluated.

**Unique INPLASY number:**

2021110001

## 1 Introduction

Coronary Heart Disease (CHD) is a common clinical cardiovascular disease and the most common type of organ pathology caused by atherosclerosis. It is more likely to occur in the elderly.^[1]^ Since the 21st century, the mortality of atherosclerotic cardiovascular disease in China has exceeded that of tumor and has become the primary disease seriously endangering people's health.^[2]^ Frequent episodes of angina pectoris are often clinically manifested.^[3]^ Difficulty breathing, fatigue, dizziness or fainting may also occur.^[4]^ The pathogenesis is heart disease caused by coronary artery endothelial injury caused by lipid deposition, resulting in coronary luminal stenosis or occlusion, and causing myocardial ischemia, hypoxia, or necrosis.^[5,6]^

The treatment principle of CHD is to improve coronary blood supply and reduce myocardial oxygen consumption, improve patient symptoms, and improve patient quality of life.^[7]^ Common treatment measures include: rest, light diet, quit smoking, limit alcohol, and take drugs that improve ischemia and relieve symptoms.^[8,9]^ In clinical practice, β-receptor antagonists, nitrate drugs, calcium channel blockers, etc. are often used to prevent myocardial infarction, and patients with severe symptoms will also use percutaneous balloon dilatation and stent implantation. treatment.^[10,11]^

Due to the limited effect of conventional western medicine treatment of coronary heart disease and many side effects.^[12]^ The patient's long-term medication compliance is insufficient. In view of the current situation, finding an effective treatment for coronary heart disease is a top priority. At present, traditional Chinese medicine has received more and more attention in the complementary and alternative therapies of patients with CHD and clinical practice.^[13–15]^

Wen-Dan Decoction (WDD) is one of the ancient prescriptions widely used in the treatment of CHD in China.^[16]^ Studies have found that WDD can lower blood lipids, improve blood rheology, reduce serum inflammatory response factors, and protect vascular endothelium.^[17–19]^ This may be related to the regulation of fat cell lipolysis pathways, cytokine-cytokine receptor interaction pathways, fat digestion and absorption pathways.^[20,21]^

## 2 Objectives

To evaluate the efficacy of WDD in the treatment of patients with CHD. And provide the latest evidence of evidence-based medicine for the clinical treatment of CHD.

## 3 Methods

### 3.1 Study protocol and registration

The protocol has been registered by us on the INPLASY website (registration number: INPLASY2021110001: https://inplasy.com/inplasy-2021-11-0001). The protocol of our study will strictly follow the Preferred Reporting Items for Systematic Review and Meta-Analysis Protocols.^[22]^

### 3.2 Study search

We will use 8 databases to search all relevant literature resources. Including four English databases PubMed, EMBASE, Web of Science, Central, and four Chinese databases China Biomedical Literature, the Wanfang Chinese digital periodical and conference database (WanFang Data), China National Knowledge Infrastructure database, and the VIP Chinese Science and Technique Journals Database (VIP). The retrieval time starts from them until August 2021. Key words include “coronary heart disease,” “wendan decoction” and “random allocation.” We will also search for ongoing or unpublished trials from the National Institutes of Health clinical registration clinical trials, the international clinical trial registration platform and the China clinical trial registration platform. PubMed's search strategy is shown in Table 1.

**Table 1:** Search strategy used in PubMed database.

Order	Search items
#1	(“Coronary Diseases”[Mesh]) OR ((((((((Disease, Coronary[Title/Abstract]) OR (Diseases, Coronary[Title/Abstract])) OR (Coronary Heart Disease[Title/Abstract])) OR (Coronary Heart Diseases[Title/Abstract])) OR (Disease, Coronary Heart[Title/Abstract])) OR (Diseases, Coronary Heart[Title/Abstract])) OR (Heart Disease, Coronary[Title/Abstract])) OR (Heart Diseases, Coronary[Title/Abstract])) OR (CHD[Title/Abstract])
#2	(((((Wen-Dan Decoction [Title/Abstract]) OR (Wen-Dan-Tang[Title/Abstract])) OR (WDD[Title/Abstract])) OR (WDT[Title/Abstract])) OR (traditional Chinese medicine[Title/Abstract])) OR (TCM[Title/Abstract])
#3	randomized controlled trial[Publication Type] OR randomized[Title/Abstract] OR placebo[Title/Abstract]
#4	#1 AND #2 AND #3

### 3.3 Inclusion criteria for research selection

#### 3.3.1 Types of studies

All randomized controlled trials of WDD in the treatment of CHD, whether blinded or unblinded.

#### 3.3.2 Type of participants

Refer to the International Society of Cardiology and Association and the World Health Organization Clinical Nomenclature Standardization Joint Task Group report “Nomenclature and Diagnostic Criteria of Ischemic Heart Disease“ for patients diagnosed with CHD.^[23]^

#### 3.3.3 Type of interventions

The treatment of the experimental group was treated with WDD on the basis of conventional drug treatment. The mode of administration is oral, and the dosage form is decoction or granule.

#### 3.3.4 Type of comparators

The control group received conventional medication or placebo treatment.

#### 3.3.5 Types of outcome measures

The main outcome indicator is total effective rate. Secondary outcome indicators include symptom score, nitroglycerin reduction rate, efficacy evaluation of electrocardiogram etc.

### 3.4 Exclusion criteria

(1) Non-randomized clinical trial literature.
(2) Animal experiments, case reports and reviews.
(3) Duplicate literature.
(4) Patients with UC and bacterial infection.
(5) Articles for which data cannot be obtained.

### 3.5 Selection of studies and data extraction

**Figure 1:**
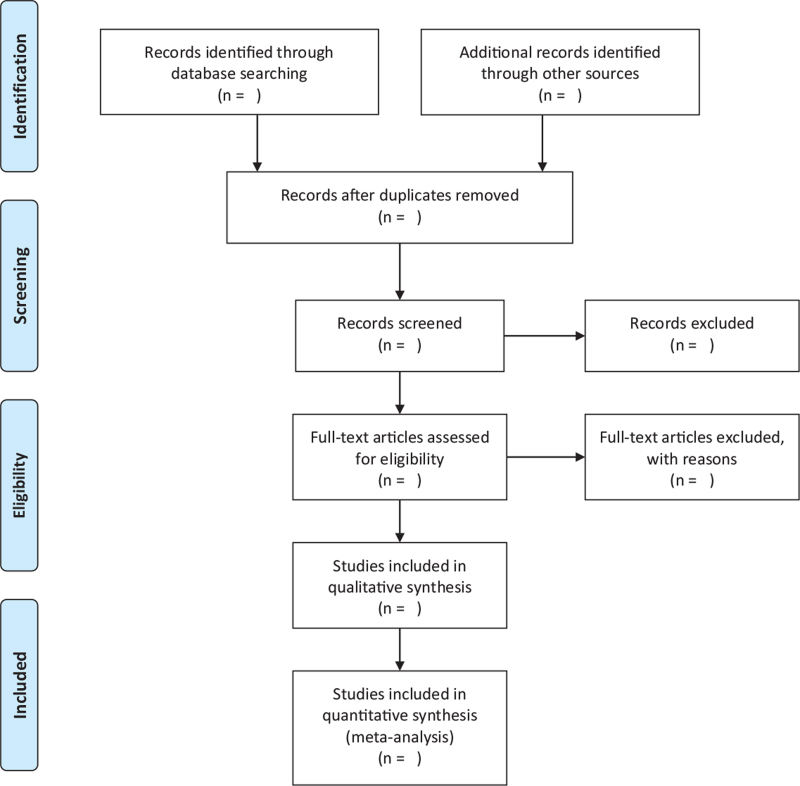
Flow chart of study selection.

Two researchers independently collated the literature. First, import all the included documents into Endnote X9 software to eliminate duplicate documents. Then read the title and abstract to eliminate documents that do not meet the requirements. Finally read the full text for screening. When two researchers have different opinions, discuss with the third researcher. We will show the specific screening process based on the Preferred Reporting Items for Systematic Review and Meta-Analysis flowchart (Fig. 1).

After completing the literature screening, the two researchers independently extracted data from the final included literature according to the pre-designed table. The extracted data includes the author's name, publication year, patient age, gender, Intervention measures (type, time, frequency, and acupuncture points) and outcome indicators. If the required information is missing or incomplete, we will email the corresponding author or first author of the original literature.

### 3.6 Risk of bias assessment

To assess the bias of the included literature, we will use the Cochrane risk assessment tool.^[24]^ Its main content includes the following 7 items: random method selection; allocation hiding; blind method, completeness of the result data; whether the evaluator is blind; selectively reporting results; other biases. The above 7 items all contain 3 options of ”yes,” ”no” and ”unclear,” and are properly evaluated by 2 researchers. If there is a disagreement during the evaluation process, find a third party for discussion.

### 3.7 Quantitative data synthesis and statistical methods

#### 3.7.1 Quantitative data synthesis

RevMan V.5.3 software will be used for data analysis and quantitative data synthesis. For continuous data, the standard mean difference with 95% confidence interval will be used for analysis. For dichotomous data, a risk ratio with 95% confidence interval will be used for evaluation.

#### 3.7.2 Assessment of heterogeneity

The *I*^*2*^ test statistic will be used to assess the heterogeneity of the included randomized clinical trials. If *I*^*2*^ <50% and *P* >.1, the results will indicate that there is no homogeneity in the study, and the fixed effects model will be used for analysis. Otherwise, it indicates that there is heterogeneity, and the reasons for the heterogeneity need to be further analyzed.

#### 3.7.3 Assessment of reporting biases

When more than 10 documents are included in the study, a funnel chart will be generated to detect reporting bias. In addition, we will also use Egger test to check the asymmetry of the funnel chart.

#### 3.7.4 Subgroup analysis and sensitivity analysis

If necessary, we will conduct a subgroup analysis based on the type of acupuncture, acupoints and other factors. In addition, we will also perform sensitivity analysis to test the stability of the results.

#### 3.7.5 Grading the quality of evidence

For consistency, risk of bias, accuracy, indirectness, and publication bias, two researchers will assess the quality of each result of the evidence according to the ” Grades of Recommendation, Assessment, Development, and Evaluation.”^[25]^ The quality of the evidence will be assessed as high, medium, low or very low.

### 3.8 Ethics and dissemination

This research is a systematic review, which results are based on published research and do not involve personal privacy. Therefore, the research does not require the review and approval of the ethics committee. We intend to publish the research results in a journal for peer review.

## 4 Discussion

CHD is a common clinical disease of the cardiovascular system. In recent years, the incidence has gradually become younger, and it is one of the main diseases that threaten humans. Due to the limited effects of conventional western medicine treatment of coronary heart disease and many side effects, patients have poor compliance with long-term medication. In China, WDD is widely used in the treatment of coronary heart disease. Studies have shown that it has an effect on protecting vascular endothelial function and reducing inflammatory reactions. Although clinical randomized controlled trials have shown that WDD is effective in the treatment of CHD, there is no systematic review and there is no international recognition. Therefore, this study conducted a comprehensive systematic review and meta-analysis of the effectiveness of WDD in the treatment of CHD. The main purpose is to provide the latest evidence for the treatment of CHD and guide clinical decision.

## Author contributions

**Conceptualization:** Xiaoyu Zhang, Yingwei Wang.

**Data curation:** Lufei Liu, Jianwei Wang.

**Formal analysis:** Hui Jiang, Yang Xiao.

**Methodology:** Lufei Liu, Jianwei Wang, Hui Jiang.

**Software:** Jing Wang, Xiaoyu Zhang.

**Supervision:** Yingwei Wang.

**Writing – original draft:** Xiaoyu Zhang, Yang Xiao, Jing Wang.

**Writing – review & editing:** Xiaoyu Zhang, Yingwei Wang, Jianwei Wang.
